# The management of an endodontically abscessed tooth: patient health state utility, decision-tree and economic analysis

**DOI:** 10.1186/1472-6831-7-17

**Published:** 2007-12-06

**Authors:** Ben Balevi, Sasha Shepperd

**Affiliations:** 1 Private Practice #306 – 805 West Broadway, Vancouver, BC, V5Z 1K1, Canada. Affiliated with the Faculty of Medicine, University of British Columbia, Room 1342-2250 Health Science Mall, Vancouver, BC V6T 1Z3, Canada; 2 Research Scientist in Evidence Synthesis, University of Oxford, Department of Public Health,. Old Road, Headington, Oxford OX3 7LF, UK

## Abstract

**Background:**

A frequent encounter in clinical practice is the middle-aged adult patient complaining of a toothache caused by the spread of a carious infection into the tooth's endodontic complex. Decisions about the range of treatment options (conventional crown with a post and core technique (CC), a single tooth implant (STI), a conventional dental bridge (CDB), and a partial removable denture (RPD)) have to balance the prognosis, utility and cost. Little is know about the utility patients attach to the different treatment options for an endontically abscessed mandibular molar and maxillary incisor. We measured patients' dental-health-state utilities and ranking preferences of the treatment options for these dental problems.

**Methods:**

Forty school teachers ranked their preferences for conventional crown with a post and core technique, a single tooth implant, a conventional dental bridge, and a partial removable denture using a standard gamble and willingness to pay. Data previously reported on treatment prognosis and direct "out-of-pocket" costs were used in a decision-tree and economic analysis

**Results:**

The Standard Gamble utilities for the restoration of a mandibular 1st molar with either the conventional crown (CC), single-tooth-implant (STI), conventional dental bridge (CDB) or removable-partial-denture (RPD) were 74.47 [± 6.91], 78.60 [± 5.19], 76.22 [± 5.78], 64.80 [± 8.1] respectively (p < 0.05). Their respective Willingness-to-Pay ($CDN) were 1,782.05 [± 361.42], 1,871.79 [± 349.44], 1,605.13 [± 348.10], 1,351.28 [± 368.62] (p < 0.05).

The standard gamble utilities for the restoration of a maxillary central incisor with a CC, STI, CDB and RPD were 88.50 [± 6.12], 90.68 [± 3.41], 89.78 [± 3.81] and 91.10 [± 3.57] respectively (p > 0.05). Their respective willingness-to-pay ($CDN) were: 1,782.05 [± 361.42], 1,871.79 [± 349.44], 1,605.13 [± 348.10] and 1,351.28 [± 368.62]. A statistical difference was found between the utility of treating a maxillary central incisor and mandibular 1st-molar (p < 0.05).

The expected-utility-value for a 5-year prosthetic survival was highest for the CDB and the STI treatment of an abscessed mandibular molar (74.75 and 71.47 respectively) and maxillary incisor (86.24 and 84.91 respectively). This held up to a sensitivity analysis when the success of root canal therapy and the risk of damage to the adjacent tooth were varied. The RPD for both the molar and incisor was the favored treatment based on a cost-utility (3.85 and 2.74 CND$ per year of tooth saved respectively) and cost-benefit analysis (0.92 to 0.60 CND$ of cost per $ of benefit, respectively) for a prosthetic clinical survival of 5-years.

**Conclusion:**

The position of the abscessed tooth and the amount of insurance coverage influences the utility and rank assigned by patients to the different treatment options. STI and CDB have optimal EUVs for a 5-year survival outcome, and RPD has significantly lower cost providing the better cost:benefit ratio.

## Background

The middle-aged adult complaining of pain from an endodontically abscessed tooth is become increasingly common in dental practice [1]. This often occurs on a tooth with a previously existing large dental restoration. The options available to manage such a clinical scenario include:

1. Saving the tooth using root canal therapy, post and core buildup (P & C), crown lengthening periodontal surgery and convention crown restoration (CC)

2. Extracting the tooth and replace it with a single-tooth-implant (STI)

3. Extracting the tooth and replace it with a conventional-dental-bridge (CDB)

4. Extracting the tooth and replace it with a removable-partial-denture (RPD)

5. Extracting the tooth and not replace it (EXO)

Having many or all of the teeth missing can be a significant health issue as it may compromise a patient's nutritional status[2]. Nevertheless, each tooth replacement therapy has its own series of sacrifices and consequences to the patient. The sacrifices include the cost of the procedure, pain and suffering associated with the treatment and the time it takes to complete treatment. Although recent evidence questions the long term deleterious effect of a single tooth edentulous space on overall dental health [3], conventional wisdom in dental practice historically mandated the establishment of a full complement of fourteen maxillary teeth in proper dental occlusion with a full complement of fourteen mandibular teeth as the "ideal" objective of dental treatment[4,5]. But what constitutes *ideal treatment *and from whose perspective should the outcome of dental treatment be judged?

When a patient assesses the treatment options available to manage an abscessed tooth, he/she must determine the value of the tooth to their over-all well-being. If it has no value, then the extraction of the tooth with no prosthetic replacement becomes the treatment of choice. If the patient values the tooth or its replacement but wants a hasty outcome, then the RPD maybe the desired option. However in order to participate in these decisions patients require relevant information at the time of making the decision.

The dental literature offers some data on the prognosis of STI, CDB, CC and RPD. A recent published meta-analysis by Salinas and Eckert (2007) estimated a favorable 5-year survival estimates of 95.1% [± 2.9] and 94.0% [± 3.3] for STI and CDB respectively[6] A Randomized-controlled-trial conducted by Creuger et al's (2005) found the 5-year survival of a crown supported by a post and core build-up to be 95.3% [± 2.4] [7]. Such a value must take into account that the likelihood of a root canal succeeding is reported about 90% [8]. Finally, the 5 year survival of a RPD was determined from single randomized-control trial to be 76.1% [± 6.3] [9].

Over the last half-century, much work has gone into developing reliable and valid methods of quantifying consumer utility [10]. A few of these methods have been applied to measuring the utility of dental services. They include: *Standard gamble*, *Willingness-to-Pay*), and, *Visual-analog-scale (VAS) or Ranking*

Utility values take on a value between zero and 100, in the case of standard gamble (often presented in units of utile) and VAS measurements, or a monetary value in the case of willingness- to-pay assessments. One can think of the utile as the proportional value judgment a patient places on a non-ideal health state relative to the ideal health state (having a value of *100%*) and the worst possible health state (having a value of *0% utile*.) For example, a utility of *75 utile *implies a health state perceived to be about 75% of ideal health

Fyffe and Kay (1992) were the first to apply the standard gamble method to dentistry [11]. They assessed the health state utility of four different tooth states using the conditions of a perfectly healthy tooth and immediate dental extraction as the upper and lower anchor points for the standard gamble. Others have since reported dental health state utilities using standard gamble [12-16].

In contrast to treatment prognosis, there is a dearth of documented patient's health state utility in the literature to the outcomes of dental prosthetics. Jacobson *et al *used a visual-analog-scale (i.e. "feeling thermometer") to evaluate the utility of 111 edentulous patients after they were treated with a conventional or implant-supported complete dentures[17]. Although, the implant-supported denture graded higher than the conventional denture treatment, no attempt was made to evaluate the perceived utility prior to undergoing the treatment. The utilities of 45 implant, 40 denture and 42 CDB patients were assessed in a survey, and the standard gamble utility for implants and CDB was 95 and 87 respectively with corresponding willingness-to-pay assessed at 4,153 and 439 UK pounds[12]. Implant treatment was the treatment of choice based on cost-effectiveness, cost-benefit and cost-utility analysis. The utility measure for the implant patients did not differentiate STI, multiple-implants or implant supported complete dentures. Also, no differentiation was made for the utility of managing anterior teeth and posterior teeth.

Mileman and van den Hout recently published the utilities of dental treatment from the perspective of the dental practitioner[16]. Using the standard gamble method, the utilities of 26 dentists were assessed for the dental management of an abscessed anterior tooth. Mean utility of 77 was found among all dentists for the restoration of a tooth with a root canal treatment followed by a dental crown. The extreme anchor reference points were *zero *for "immediate extraction and bridge" and 100 for "composite restoration in a vital tooth."

We extended this technique by measuring patients' utilities and ranking preferences for the management of an endodontically abscessed mandibular molar and maxillary incisor treatment options. We used these values and published survival data in a decision tree analysis to determine the most favored option. We analyzed each treatment's prognosis against its utility for the sake of making the *"best" *decision in the face of uncertainty.

## Methods

### Part 1 – Assessing patient preferences and health-state utilities

A convenience sample of school teachers from the of Vancouver (British Columbia, Canada) area were identified by *"snowballing"*[18] for potential participants to this study. This involved asking participants to help recruit similar participants in this target group to enter the study. Those interested in participating were provided with information describing the investigation and asked to sign a form consenting to participate in the study. Teachers were recruited as they represent the average middle income dental patient, and often have access to private dental insurance. In addition, teachers were more likely to understand the concepts presented in this study because of their level of education.

Participants were given a package of blank answer sheets at the start of the interview. They were told to imagine that they had an abscessed tooth. The investigator (BB) then described the five common treatment options currently available to them. They included; (i) root canal treatment/post & core and restored with a *conventional-crown *(CC), (ii) extraction and restored with a single-*tooth-implant *(STI), (iii) extraction and restored with a *conventional-dental bridge *(CDB), (iv) extraction and restored with a *removable-partial-denture *(RPD) and (v) extraction with *no restorative prosthesis *(EXO).

The clinical procedure of each treatment option was described in detail with the help of models, detailed written information and patient education software (Optio Dentistry^®^, Optio Publishing Co., 1668 Barrignton Street, Suite 40, Halifax, Nova Scotia, B3J 2A2, Canada). Two methods were used to obtain the participant's utilities for each procedure.

#### Standard-gamble utility

**Figure 1:**
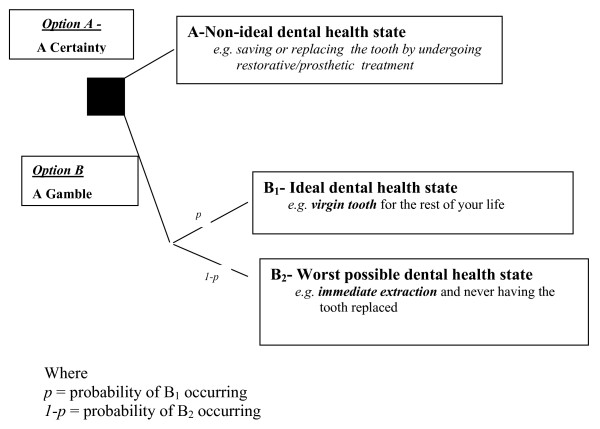
Standard gamble decision algorithm.

The design of the standard gamble method used was modelled after Fyffe and Kay [11] (Figure 1).

The gamble's upper anchor was defined as the hypothetical re-establishment of the abscessed tooth back to a perfectly healthy virgin state, with the lower anchor point defined as the immediate extraction of the abscessed tooth and the inevitability of living with the edentulous space for the rest of their life.

#### Willingness-to-pay

A bidding algorithm similar to that described by Matthew's *et al *was used to assist participants determine their willingness-to-pay threshold for each treatment option before they accepted losing the tooth and living with the edentulous space for the rest of their life[19]. The highest bid and lowest bid were set at 3,600 $CDN and nothing respectively. This method lends itself well to evaluating dental treatments since often a direct exchange of money occurs between the patient receiving and the dentist delivering the service [12,19-23].

#### Preference choice

The subjects were asked to rank the five treatment options in order of preference, with the most and least preferred treatment ranked #1 and #5 respectively. They were to assume complete cost for the treatment (i.e., 0% dental insurance coverage). Subjects repeated this ranking exercise assuming they had dental insurance that would cover 25%, 50%, 75% and 100% of the total treatment.

#### Participants' comments

Those participating were asked to provide written responses to the following three open ended questions:

1 What factors will determine your decision amongst a list of dental treatments?

2 What difficulties did you have participating in this study?

3 What value did you gain from participating in this study?

### Sample size calculation

A sample size of 42 individuals was calculated. This calculation was based on an 80% power and an alpha of 0.05, to detect a difference of 0.1 utility points measured by standard gamble and an estimated standard deviation of 0.16 taken from Milman and van de Hout (2003) [16]. A 10% utility difference was arbitrarily selected as the differences expected to be observed between choices. This expectation was based on previously reported studies using standard gamble to measure dental health utilities [11,13,14,16,24].

### Data analysis

All statistical analysis of the data was carried out using SPSS (version 11), and the decision analysis was carried out using TreeAge Pro 2007 Suite^® ^(TreeAge Software Inc, Williamstown MA 01267). An ANOVA was used to test the null hypothesis that no statistical difference existed in the standard gamble and willingness-to-pay utility between the four restorative treatment options. If the null hypothesis was rejected then a Tukey post-hoc multiple comparison test was used to identify which treatment options differed. A Pearson correlation statistic assessed the strength of any possible association between the utility measured via the standard gamble and willingness-to-pay technique.

All statistical significance were tested at the p < .05 level.

### Part 2 – Decision-tree construction and analysis

**Figure 2:**
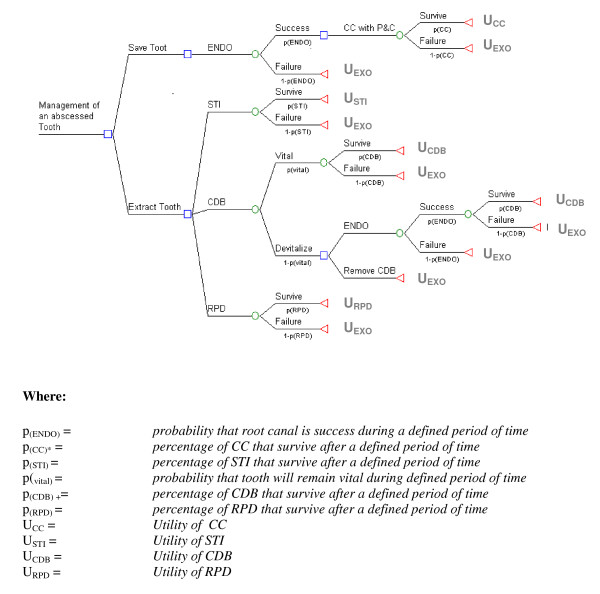
**Decision tree for the management of an abscessed tooth**. (The square box signifies the *decision node*. It is at this point in the decision tree that the decision maker is asked to make a choice. What follows are the circular *chance nodes*. Branching from these chance nodes are possible consequences, as represented by the probabilities of a positive and negative outcome. The *end node*, as depicted by the triangle, indicates that the decision has been completed. At this point, all uncertainties and utilities associated with each decision have been incurred. In other words, the decision maker understands that once they commit to a decision, they accept its associated uncertainty and utility and thus consequences.)

Figure 2 is a generic decision tree for the management of an abscessed tooth. We used a time-line of 5-years, and assumed that the teeth adjacent to the abscessed tooth were vital. Such a time frame was used because; (1) prognostic data of longer than five years are difficult to find and (2) private insurance companies will often cover the cost of prosthetic replacement every five years.

The quantitative interpretation of a decision tree is determined by the *expected utility value *(EUV) of each decision. This is simply the weighted average of all probabilities and utilities associated with each branch of the decision node (i.e., *folding back the tree*) [25,26]. Decision-tree analysis is based on the grounds that the "reasonable" decision maker, accepting that they live in a world of uncertainty, seeks to make an *a priori *choice (i.e., gamble) that maximizes their EUV.

The risk of irreversible endodontic damage after conventional crowning of a vital tooth was previously reported[27]. Five-year survival probabilities for each treatment are given in Table 1.

**Table 1:** Probability value of survival and success

**Variable**	**Value**	**Reference**
p(ENDO) =	*.90*	Friedman and Mor (2004) [8]
p(CC) =	*.953*	Creuger et al (2005) [7]
p(STI) =	*.951*	Salinas and Eckert (2007) [6]
p(vital) =	*.976*	Habsha (1998) [27]
p(CDB) =	*.940*	Salinas and Eckert (2007) [6]
p(RPD) =	*.761*	Kapur et al (1994) [9]

#### Cost-utility analysis (CUA)

Cost utility analysis attempts to relate the cost of each treatment with the patient's perceived change to their quality of life. We assigned cost using the direct *out-of-pocket *costs that the patient or insurer pays to the dentist. We transformed standard gamble utilities into a temporal construct of Quality-of-Tooth-Years (QLTY) given by the following formula [28].

*QLTY *= [*EUV*] × [*years of survival*]

Cost-Utility ratios were calculated by dividing the direct cost by the QLTY[25,29].

#### Cost-benefit analysis (CBA)

A cost-benefit-analysis attempts to compare the financial cost of treatment options with the *benefit *of its outcome. In this case the benefit, or utility, is given in monetary units by *willingness-to-pay*. Cost-Benefit was determined by dividing the direct cost by its expected *willingness-to-pay *value (i.e., benefit) for each treatment[25,29].

### Sensitivity analysis

This decision-tree model considers the trade-offs between risks of a failed root canal therapy and/or irreversible endodontic damage to a dental bridge's abutment teeth against the benefit of a fixed prosthesis which avoids the invasive surgeries of a dental extraction or dental implant therapy. Sensitivity analysis was carried out changing the probability of the success of root canal therapy and the risk of irreversible damage to a dental bridge's abutment teeth

## Results

### Patient preferences and health-state utilities

Forty teachers consented to participate in this study. The mean age of the participants was 48.9 (± 2.55) years. Females made up 75% of all participants. All but one participant (97.5%) reported visiting a dentist within the last twelve months. A sizable majority (31/40,77.5%) reported experiencing toothache. Twenty-three (57.5%) reported missing at least one tooth, 25/40 (62.5%) undergoing root canal therapy and 11/40 (27.5%) gum surgery. Thirty (70%) and 9/40 (22.5%) were fitted with a dental crown or bridge respectively. Few reported wearing a removable denture (4/40, 10%) or undergoing dental implant treatment (3/40,7.5%).

Eighty percent (32/40) cited financial reasons as a factor in determining their dental treatment decisions. Other factors important in their decision included: esthetics, durability and intrusiveness of the dental treatment option. The vast majority of the participants claimed that they had no difficulties understanding the concepts of this survey. The utilities for the standard gamble and willingness-to-pay for both the mandibular 1^st ^molar and maxillary central incisor are given in Table 2.

**Table 2:** Utility measurement for the management of an abscess mandibular 1^st ^molar and maxillary central incisor*

**Tooth**	**Utility Measurement**	**Treatment option**				**Statistic (F_3,156_)**	**Sig**
				
		**CC**	**STI**	**CDB**	**RPD**		
**Molar**	**Standard gamble **(utile)	74.47 [6.91]	78.60 [5.19]	76.22 [5.78]	64.80 [8.10]	3.424	P < .019
	**Willingness-to-pay **(SCDN)	1,782.05 [361.42]	1,871.79 [349.44]	1,605.13 [348.10]	1,351.28 [368.62]	1.779	p < .153
**Incisor**	**Standard gamble **(utile)	88.50 [6.12]	90.68 [3,41]	89.78 [3.81]	91.10 [3.57]	0.284	p < .837
	**Willingness-to-pay **(SCDN)	2,5552.50 [333.07]	2,515.00 [315.07]	2,345.00 [336.28	2090.00 [407.82]	1.484	p < .221

*(N = 40, 95% CI given in [brackets])

#### Mandibular 1^st ^molar

The standard gamble utility is highest for the STI (78.60 ± 5.19) while that of the RPD (64.80 ± 8.1) is more than ten points below the utility of the other treatment options [p < .017].

Although the willingness-to-pay utilities were higher for the fixed restorative treatment compared with the RPD options, no statistical significance was found.

A weak positive correlation exists between the standard gamble and willingness-to-pay utility (n = 160; Pearson's *r *= 0.196; P = .013).

**Figure 3:**
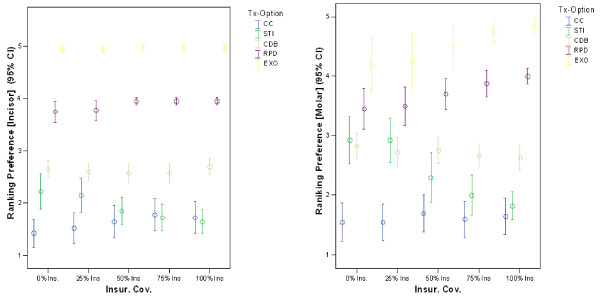
**Preference ranking for each treatment option as a function of level of dental insurance coverage**. (with 95% CI where 1 = most preferred, and, 5 = least preferred)

Ranking between treatment options – including extraction of tooth without prosthetic replacement) as a function of insurance coverage is presented graphically in Figure 3. A general positive trend in the selection of STI occurs with increased insurance coverage. Also, a dramatic negative effect on the selection of extraction occurs with increased insurance converge. Finally, the confidence intervals become progressively smaller as the financial responsibility on the patients lessens.

#### Maxillary central incisor

The standard gamble utilities for the management of an abscessed maxillary central incisor are similarly high (i.e., approx 90 utilities) for all treatment options, with no statistical significance between them.

Similarly, participants' willingness-to-pay were equally high for the restoration of an anterior tooth. Once again, no significant difference was observed between the treatment options

A positive correlation existed between the standard gamble and willingness-to-pay utility (n = 160; Pearson's *r *= 0.217; p = .006).

A slight positive trend in the ranking of STI and a slight negative trend in the ranking of CC occurs with increased insurance coverage (Figure 3). The other three options do not appear to be influenced by insurance coverage. A consistent low ranking and narrow confidence interval is evident for the RPD and EXO option.

### Decision-tree and economic analysis

**Figure 4:**
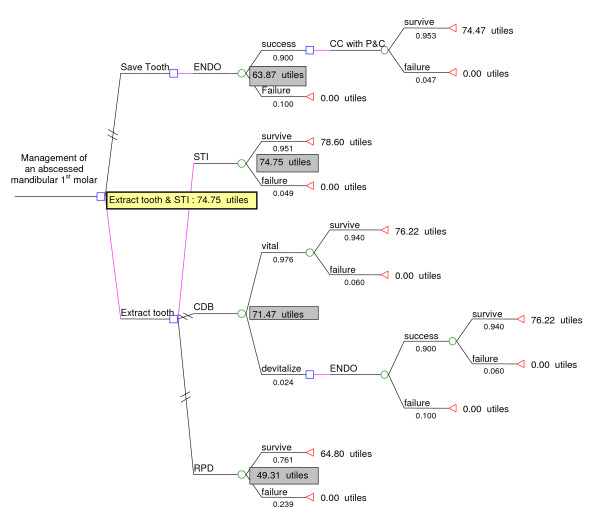
*Folding-back *analysis of decision tree with associated expected-utility-values for the management of an abscessed mandibular 1^st ^molar.

Figure 4 depicts, as an example, a detailed diagrammatic "folding back" analysis of the decision-tree, with its associated EUV, for the management of an abscessed mandibular 1^st ^molar. Table 3 presented the detailed folding back calculation of the weighted standard gamble and willingness-to-pay utility (referred to as the EUV) for a detailed decision tree analysis mandibular molar and maxillary incisor

**Table 3:** Folding-back decision tree analysis calculation

**Tooth**	**Utility measurement**	**Treatment option**	**EUV calculation**^α^
**Molar**	**Standard gamble**	CC	[74.47 × 0.900 × 0.953]+ [(0.00 × 0.900 × 0.047)]+ [0.00 × 0.100] = **63.87 utile**
		STI	[78.60 × .951] + [0.00 × 0.049] = **74.75 utile**
		CDB	[76.22 × 0.976 × 0.940] + [0.00 × 0.976 × 0.940] + [(76.22 × 0.940 × 0.900) + (0.00 × 0.940 × 0.900)] + (0.00 × 0.100)] × 0.024 = **71.47 utile**
		RPD	= [64.80 × 0.761] + [0.00 × 0.239] = **49.31 utile**
	**Willingness-to-pay**	CC	[$1,782.05 × 0.900 × 0.953]+ [($0.00 × 0.900 × .047)]+ [$0.00 × 0.100] = **1,528.46 $CDN**
		STI	[$1,871.78 × 0.951] + [$0.00 × 0.049] = **1,780.07 $CDN**
		CDB	[$1,605.13 × 0.976 × 0.940] + [$0.00 × 0.976 × 0.940] + [($1,605.13 × 0.940 × 0.900) + ($0.00 × 0.940 × 0.900)] + ($0.00 × 0.100)] × 0.024 = **$1,505.20 $CDN**
		RPD	[$1,351.28 × 0.761] + [$0.00 × 0.239] = **1,028.32 $CDN**
**Incisor**	**Standard gamble**	CC	[88.50 × 0.900 × .953]+ [(0.00 × 0.900 × .047)]+ [0.00 × 0.100] = **75.91utile**
		STI	[90.68 × .951] + [0.00 × .049] = **86.24 utile**
		CDB	[89.78 × 0.976 × 0.940] + [0.00 × 0.976 × 0.940] + [(89.78 × 0.940 × 0.900) + (0.00 × 0.940 × 0.900)] + (0.00 × 0.100)] × 0.024 = **84.91 utile**
		RPD	[91.10 × 0.761] + [0.00 × 0.239] = **69.33 utile**
	**Willingness-to-pay**	CC	[$2,552.50 × 0.900 × 0.953]+ [($0.00 × 0.900 × 0.047)]+ [$0.00 × 0.100] = **2,189.28 $CDN**
		STI	[$2,515.00 × 0.951] + [$0.00 × 0.049] = **2,391.76 $CDN**
		CDB	[$2,345.00 × .976 × 0.940] + [$0.00 × .976 × 0.940] + [($2,345.00 × 0.940 × 0.900) + ($0.00 × 0.940 × 0.900)] + ($0.00 × 0.100)] × 0.024 = **2,199.10**$**CDN**
		RPD	[$2,090.00 × 0.761] + [$0.00 × 0.239] = **1,590.49 $CDN**

α-The expected-utility-value is the weighted average of the respective standard gamble and willingness-to-pay utilities for the respective dental of an abscessed 1^st ^mandibular molar and maxillary central incisor

The STI offers the highest EUV of 74.75 and 86.24 utile for the management of an abscessed molar and incisor respectively. These values are slightly higher than the EUV for the CDB (71.47 utile for the molar, 84.19 utile for the incisor), and considerably higher than the EUV for the CC (63.87 utile for the molar, 75.91 utile for incisor) and RPD (49.31 utile for the molar and 69.33 utile for the incisor).

**Figure 5:**
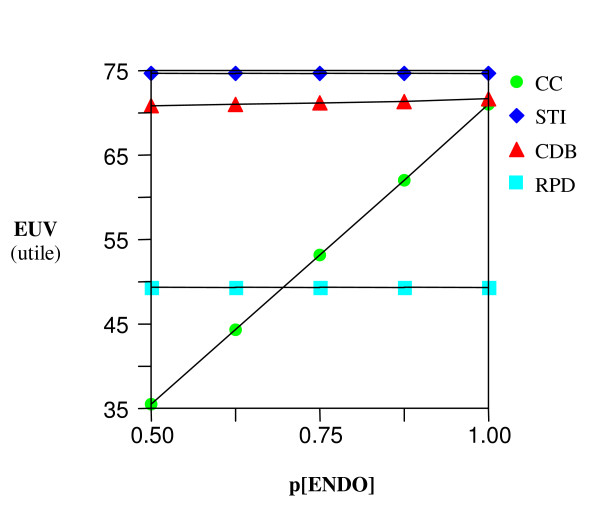
**Sensitivity analysis – Molar**. EUV Vs. varying probability of success of root canal therapy.

**Figure 6:**
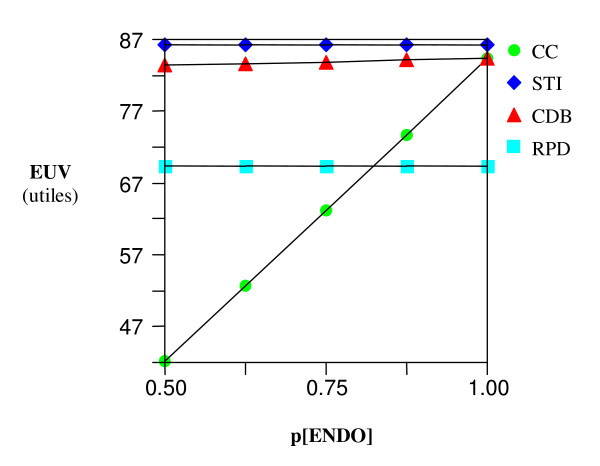
**Sensitivity analysis – Incisor**. EUV Vs. varying probability of success of root canal therapy

A sensitivity analysis on the effect of changes on the probability of successes of a root canal treatment appears to have the most noticeable effect on the EUV of the CC (Figures 5 and 6) yet its EUV never reaches the optimal threshold of the STI and CDB options. Also, no threshold was ever met on a sensitivity analysis of the EUV as a function of the risk of endodontic damage to the abutment tooth of a CDB.

### Cost-utility analysis

The Cost-Utility ratios for each tooth and respective treatment options are presented in Table 4. A 5-year Cost-Utility comparison of the four treatment options shows the RPD to be the most efficient service costing only 3.85$CND and 2.74 $CND per year of tooth survived per utile-yr of tooth survival per utility for the molar and incisor respectively and. The STI is the least efficient with a cost-utility of just under twice that of the RPD.

**Table 4:** Cost-Utility-Analysis AND Cost-Benefit Analysis (5-year).

			**Cost Utility**			**Cost-Benefit**	
			
**Tooth**	**Treatment Option**	**Treatment cost ($CND)**	**EUV (utile)**	**QATY^β ^(utile-yr)**	**Cost: Utility^β ^(CND$ per utile-yr of tooth survival)**	**Expected Benefit Value (CDN$)**	**Cost: Benefit^β ^($ paid per dollar of benefit received)**
**Molar**	**CC**	2,200	63.87	319.35	6.90	1,528.46	1.44
	**STI**	3,600	74.75	378.75	9.51	1,780.07	2.02
	**CDB**^γ^	2,620	71.47	357.35	7.73	1,505.20	1.74
	**RPD**	950	49.31	246.55	3.85	1,028.32	0.92
**Incisor**	**CC**	2,000	75.91	379.55	5.27	2,189.28	0.91
	**STI**	3,600	86.24	431.20	8.35	2,391.76	1.51
	**CDB**^γ^	2,608	84.91	424.55	6.13	2,199.10	1.19
	**RPD**	950	69.33	346.65	2.74	1,590.49	0.60

β-QATY = EUV × 5 years. Cost:Utility = Treatment cost/QATY, Cost:Benefit = Treatment cost/Expected Benefit Value.

γ – These cost of CDB option takes into account the 2.4% risk that one of the abutment teeth will incur the cost of root canal therapy in the future.

### Cost-benefit analysis

A cost-benefit-analysis attempts to compare the financial cost of treatment options with the benefit of its outcome. In this case the benefit, or utility, is given in monetary units based on the respective treatment option's *Willingness-to-pay *EUV. The cost-benefit-ratio for each option is given in Table 4.

From a 5-year perspective, only the RPD comes out ahead offering more dollar of benefit than dollar of cost paid regardless of tooth affected. The other three options require a greater than one dollar payout per dollar of perceived benefit received. However, the costs of the other treatments on the incisor are more closely aligned with the perceived benefit received as compare to the case of the molar. This reflects the greater perceived value people put on restoring an anterior tooth compared to a posterior tooth.

## Discussion

We determined the health state utilities for the management of a posterior and anterior tooth abscesses in a sample of teachers. Teachers were recruited to this study as they were likely to understand the concepts of the standard gamble and willingness-to-pay techniques, and, are representative of a typical average income Canadian with private dental insurance. The teachers recruited to this study were similar to the average Canadian population in terms of age and salary. Although the sample was predominately women this is consistent with the gender profile of the teaching profession in the British Columbian public school [30]. The average age of teachers in British Columbia is 48 years with an average income of 58,688 $CDN. This is typical of the average adult Canadian with an age of 49.6 years and household income of 59,000 $CDN [31,32]. Although women are slightly over represented in this study, the over representation of woman in the teaching profession is comparable to the higher utilization of healthcare, including dental care, by woman than men [33,34].

The *snowballing *sampling technique is an acceptable method especially to explore such complex issues as health-state utilities [18]. Nevertheless, it potentially introduces sampling bias with the inclusion of friends with similar beliefs, preferences and dental histories in the study.

Individual patient interviews were carried out to resemble a typical patient-dentist interaction in the dental office. Patients found the standardized visual, written and verbal descriptions of all treatment options informative and easy to understand. No participant expressed confusion with this aspect of the study.

Two common methods of measuring health-state-utilities were used in this study. There is a subtle difference between these two methods and, as such, they don't perfectly correlate. Standard Gamble is a choice based method that plays on the concept of risk. The use of standard gamble in this dental utility assessment defines two easily conceptualized upper and lower anchor points that can be imagined to be real by all people. For this reason, standard gamble lends itself well in measuring dental health state utilities and probably is less influenced by a "sure thing" of a successful prosthetic treatment option then it is influenced by the merit of the dental outcome itself [11,35].

*Willingness-to-pay *places the individual in the perspective of a purchaser and not a gambler [36]. Since money is easier to conceptualize then a hypothetical probability, Willingness-to-pay is more sensitive to changes in the description of the outcome then standard gamble. For this reason, standard gamble utility reflected a 25% difference between molar and incisor utility as compared to a more than 50% difference in the willingness-to-pay assessment.

The standard gamble and willingness-to-pay techniques used in this study appear to be effective at measuring the value of the dental outcome of having a tooth (CC) or some semblance of one (STI, CDB, RPD). This is clearly demonstrated when it comes to the management of a tooth in the esthetic zone, where the utility of all the treatment options, including the RPD, had equally high value. When the anchor points are 'perfectly healthy tooth' and 'no tooth', standard gamble and willingness-to-pay are effective methods of assessing health-state-utilities of an edentulous space. This study demonstrates that patients will not tolerate a missing maxillary central incisor compared to a missing mandible molar. This is further supported by the results of the ranking assessment, where just under one-in-five participants were willing to live with a missing posterior tooth if they had to pay for the treatment. No participants were willing to live with a missing anterior tooth regardless if it was an out-of-pocket expense. The confidence intervals include a narrow range of values, even with the relatively small sample size of this study. The narrow confidence intervals generated in the ranking technique further confirmed the validity of this approach in assessing a patients' preference amongst a series of choices.

Responses to the question on the first open-ended qualitative question demonstrate the significance of financial constraints on an individual's decision. The standard gamble and willingness-to-pay did not require the participants to consider this constraint in their response. Also, although the extraction and the RPD were the least costly of the five treatment options, they were still ranked as the lowest preferred choice. Cost did impact on the preference of the more complex and costly STI and CDB treatment options. As the cost of the CDB became comparable to that of the STI, the later options became the preferred treatment of choice. Therefore, from both the qualitative response of the open-ended question and the qualitative preference-ranking data, it appears that type of treatment decision depends on insurance coverage. This was particularly evident when it came to managing a molar tooth, where the ranking of extraction with no further treatment was inversely related to the amount of insurance coverage, demonstrating the discrepancy of dental care by socioeconomic gradient [37]. Nevertheless, the results of this study demonstrate the multifactorial aspect of patient's treatment preferences. A qualitative/quantitative hybrid research design could possibly elucidate the significance of each factor.

It is difficult to compare the result generated in this study with those of Jacobson *et al.*[17]. In that study, a visual-analog-scale method (VAS) was used whereas none was used in this study. Milenam and van der Hoote's study surveyed dentists and not patients[16]. Also, their study defined different upper and lower anchor points thus making comparison with this study difficult. Stella Kwan's study investigated post-treatment utilities of patients who already underwent dental implants, CDB and denture therapy [12]. Also, contrary to the findings in this study, she found that there was no difference in the utility found between anterior and posterior teeth. This may be due to differing definitions of the utility anchor and different study populations.

What constitutes the best decision will depend on the perspective of the decision maker. From the perspective of the individual patient a decision tree analysis determined that the STI and CDB had the greatest utility as treatments to restore an abscessed molar and incisor. This decision held up to a sensitivity analysis that considered two commonly recognized risks: root canal failure and irreversible endodontic damage to the CDB's abutments.

In the clinical case where CC is not an option, then either the STI or CDB will almost equally optimize the expected utility value of restoring an abscessed tooth expected to last at least five years.

We measured the EUVs assessed perceived value against prognosis and with no consideration of the financial cost to the patient. In this context the optimal decision generated by this utility analysis agrees with previously reported patients' preference ranking of identical treatment options in the context of no direct financial burden. The consistency between these two approaches supports the validity of the ranking method.

Economic cost analysis adds a third dimension to decision analysis. Confronted with a limited amount of financial resources, those paying must consider the cost of their decisions. The cost analysis favors the selection of the RPD because of its considerably lower initial cost. This is obvious from the point of view of cost-benefit-analysis, where, only the RPD had a ratio of less than one. A ratio above one essentially means that it cost more than a dollar for each dollar of benefit received – as perceived by weighted willingness to pay calculation. Except for the risk of future root canal therapy on the abutment teeth of a CDB, the economic analysis presented here does not take into account the cost of maintenance and repair to the other prosthesis in the event they fail. For example, a denture places a significant risk of future damage to the remaining teeth in the mouth as compared to a fixed dental prosthesis [38]. Although the RPD offers the lowest initial capital cost, the future cost of repair, maintenance and even replacement may negatively affect the long-term economic value of this option as compared to the other "fixed" restorative options

A limitation of this decision analysis is the limited time frame of 5 years, especially when you consider that dental caries is the most common cause of long term prosthetic failure in the long term [38,39]. Such confounding factor may show STI to have a more favorable EUV and cost-benefit ratio for time frames of 10–20 years [6]. This can only be determined when longer-term prognostic assessments of the treatment options considered in this analysis are available in the literature.

Decision-trees can be an effective approach at guiding dental health care to make choices amongst treatment alternatives [25,40]. Rohlin and Mileman (2000) – in a systematic review of the literature – found the application of decision tree analysis in dentistry to be limited and significantly lags behind medicine in guiding the efficient delivery of oral healthcare [41]. Although a comprehensive decision tree analysis for the management of an incipient occlusal carious lesion is currently available in the literature [42], this is the first such analysis on the four common treatment options available for the management of an abscessed tooth.

## Conclusion

In conclusion, the position of the abscessed tooth and the amount of insurance coverage influences the utility and rank assigned by patients to the different treatment options. Decision analysis is a helpful clinical tool in a world of varied patient preferences and uncertain dental treatment outcomes. Nevertheless, the applicability and validity of such an analysis in dentistry depends on the quality of data plugged into its model. In the decision analysis presented here STI and CDB have optimal EUVs for a 5-year survival outcome. The RPD provides the best cost:benefit ratio because of a significantly lower cost. Future research is needed to evaluate and validate treatment prognosis of dental care of longer than 5 years and measuring the perceived patient health state utility of dental outcomes.

## Competing interests

The author(s) declare that they have no competing interests.

## Authors' contributions

BB conceived the aim and objective, experimental design, carried out the experiments, analyzed and interpreted the data and wrote the manuscript. SS contributed to the design of the study, the interpretation of the data and writing the paper.

## Pre-publication history

The pre-publication history for this paper can be accessed here:


